# Retraction Note: Oral health-related quality of life in rheumatoid arthritis: a comparative analysis

**DOI:** 10.1186/s41927-024-00402-w

**Published:** 2024-07-16

**Authors:** Amirhossein Parsaei, Aida Mehdipour, Hamidreza Ghadimi, Ashkan Mohammadi Kooshki, Parisa Shajari, Maryam Masoumi, Pouya Torabi, Hossein Azizi, Behnam Amini, Hanie Karimi, Hojat Dehghanbanadaki, Mohammad Aghaali, Soroush Moradi

**Affiliations:** 1 Faculty of Medicine, Tehran University of Medical Sciences, Tehran, Iran; 2 Qom Dental School, Qom University of Medical Sciences, Qom, Iran; 3 School of Medicine, Qom University of Medical Sciences, Qom, Iran; 4 School of Medicine, Iran University of Medical Science, Tehran, Iran; 5 Student Research Committee, School of Dentistry, Qom University of Medical Sciences, Qom, Iran; 6 Clinical Research of Development Center, Shahid Beheshti Hospital, Qom University of Medical Sciences, Qom, Qom Iran; 7 Endocrinology and Metabolism Research Center, Endocrinology and Metabolism Clinical Sciences Institute, Tehran University of Medical Sciences, Tehran, Iran; 8 Department of Community Medicine, School of Medicine, Qom University of Medical Sciences, Qom, Iran

**Retraction Note: BMC Rheumatology (2022) 6:61**.

10.1186/s41927-022-00292-w.

The Editor has retracted this article. Concerns were raised about similarities in the patients’ cohorts and data between this article and a previously-published paper by some of the same authors [1] that was also under submission at the same time, making the article redundant. The authors provided raw data which did not alleviate the concerns. Additionally, the authors did not provide evidence that an appropriate ethics approval had been received before the commencement of this study.

Hanie Karimi and Pouya Torabi agree to this retraction. Maryam Masoumi did not state explicitly whether they agree to this retraction. Amirhossein Parsaei, Aida Mehdipour, Hamidreza Ghadimi, Ashkan Mohammadi Kooshki, Parisa Shajari, Hossein Azizi, Behnam Amini, Hojat Dehghanbanadaki, Mohammad Aghaali, and Soroush Moradi did not respond to correspondence from the Editor about this retraction.
